# Supplementary material for: Unveiling the Bioactive Potential of the Invasive Jellyfish Phyllorhiza punctata Through Integrative Transcriptomic and Proteomic Analyses
Source: Biomolecules. 2025 Aug 4;15(8):1121. doi: 10.3390/biom15081121 (PMC12383608; doi:10.3390/biom15081121)
Supplement: Supplementary file 1 [file biomolecules-15-01121-s001.zip › Suplementary Material/FileS3/PEPFOLD4/XP_065063860_11043/clusters.html]

 PEP-FOLD clusters


Download archive of all models  here   

|  |  |  |
| --- | --- | --- |
| # | file | sOPEP |
| # cluster 1 - Size: 24 |  |  |
|  | XP\_065063860\_11043-model1 | -12.2913 |
|  | XP\_065063860\_11043-model1.1 | -12.2913 |
|  | XP\_065063860\_11043-model1.2 | -12.2913 |
|  | XP\_065063860\_11043-model1.3 | -12.2913 |
|  | XP\_065063860\_11043-model1.4 | -12.2913 |
|  | XP\_065063860\_11043-model1.5 | -12.2913 |
|  | XP\_065063860\_11043-model1.6 | -12.2913 |
|  | XP\_065063860\_11043-model1.7 | -12.2913 |
|  | XP\_065063860\_11043-model1.8 | -12.2913 |
|  | XP\_065063860\_11043-model1.9 | -12.218 |
|  | XP\_065063860\_11043-model1.10 | -12.218 |
|  | XP\_065063860\_11043-model1.11 | -12.218 |
|  | XP\_065063860\_11043-model1.12 | -12.218 |
|  | XP\_065063860\_11043-model1.13 | -12.218 |
|  | XP\_065063860\_11043-model1.14 | -12.218 |
|  | XP\_065063860\_11043-model1.15 | -12.218 |
|  | XP\_065063860\_11043-model1.16 | -12.218 |
|  | XP\_065063860\_11043-model1.17 | -12.218 |
|  | XP\_065063860\_11043-model1.18 | -12.218 |
|  | XP\_065063860\_11043-model1.19 | -12.218 |
|  | XP\_065063860\_11043-model1.20 | -12.218 |
|  | XP\_065063860\_11043-model1.21 | -12.1424 |
|  | XP\_065063860\_11043-model1.22 | -12.1424 |
|  | XP\_065063860\_11043-model1.23 | -12.1424 |
| # cluster 2 - Size: 48 |  |  |
|  | XP\_065063860\_11043-model2 | -12.2301 |
|  | XP\_065063860\_11043-model2.1 | -12.2301 |
|  | XP\_065063860\_11043-model2.2 | -12.2301 |
|  | XP\_065063860\_11043-model2.3 | -12.2301 |
|  | XP\_065063860\_11043-model2.4 | -12.2301 |
|  | XP\_065063860\_11043-model2.5 | -12.2301 |
|  | XP\_065063860\_11043-model2.6 | -12.2301 |
|  | XP\_065063860\_11043-model2.7 | -12.2301 |
|  | XP\_065063860\_11043-model2.8 | -12.2301 |
|  | XP\_065063860\_11043-model2.9 | -12.2301 |
|  | XP\_065063860\_11043-model2.10 | -12.2301 |
|  | XP\_065063860\_11043-model2.11 | -12.2301 |
|  | XP\_065063860\_11043-model2.12 | -12.2301 |
|  | XP\_065063860\_11043-model2.13 | -12.2301 |
|  | XP\_065063860\_11043-model2.14 | -12.2301 |
|  | XP\_065063860\_11043-model2.15 | -12.2301 |
|  | XP\_065063860\_11043-model2.16 | -12.2301 |
|  | XP\_065063860\_11043-model2.17 | -12.2301 |
|  | XP\_065063860\_11043-model2.18 | -12.2301 |
|  | XP\_065063860\_11043-model2.19 | -12.2301 |
|  | XP\_065063860\_11043-model2.20 | -12.2301 |
|  | XP\_065063860\_11043-model2.21 | -12.2301 |
|  | XP\_065063860\_11043-model2.22 | -12.2301 |
|  | XP\_065063860\_11043-model2.23 | -12.2301 |
|  | XP\_065063860\_11043-model2.24 | -12.2301 |
|  | XP\_065063860\_11043-model2.25 | -12.2301 |
|  | XP\_065063860\_11043-model2.26 | -12.2301 |
|  | XP\_065063860\_11043-model2.27 | -12.2301 |
|  | XP\_065063860\_11043-model2.28 | -12.2301 |
|  | XP\_065063860\_11043-model2.29 | -12.2301 |
|  | XP\_065063860\_11043-model2.30 | -12.2301 |
|  | XP\_065063860\_11043-model2.31 | -12.2301 |
|  | XP\_065063860\_11043-model2.32 | -12.2301 |
|  | XP\_065063860\_11043-model2.33 | -12.2301 |
|  | XP\_065063860\_11043-model2.34 | -12.2301 |
|  | XP\_065063860\_11043-model2.35 | -12.2301 |
|  | XP\_065063860\_11043-model2.36 | -12.2301 |
|  | XP\_065063860\_11043-model2.37 | -12.2301 |
|  | XP\_065063860\_11043-model2.38 | -12.2301 |
|  | XP\_065063860\_11043-model2.39 | -12.2301 |
|  | XP\_065063860\_11043-model2.40 | -12.2301 |
|  | XP\_065063860\_11043-model2.41 | -12.2301 |
|  | XP\_065063860\_11043-model2.42 | -12.2301 |
|  | XP\_065063860\_11043-model2.43 | -12.2301 |
|  | XP\_065063860\_11043-model2.44 | -12.2301 |
|  | XP\_065063860\_11043-model2.45 | -12.2301 |
|  | XP\_065063860\_11043-model2.46 | -12.2301 |
|  | XP\_065063860\_11043-model2.47 | -12.2301 |
| # cluster 3 - Size: 5 |  |  |
|  | XP\_065063860\_11043-model3 | -12.2058 |
|  | XP\_065063860\_11043-model3.1 | -12.2058 |
|  | XP\_065063860\_11043-model3.2 | -12.2058 |
|  | XP\_065063860\_11043-model3.3 | -12.2058 |
|  | XP\_065063860\_11043-model3.4 | -12.2058 |
| # cluster 4 - Size: 16 |  |  |
|  | XP\_065063860\_11043-model4 | -12.1978 |
|  | XP\_065063860\_11043-model4.1 | -12.1978 |
|  | XP\_065063860\_11043-model4.2 | -12.1978 |
|  | XP\_065063860\_11043-model4.3 | -12.1978 |
|  | XP\_065063860\_11043-model4.4 | -12.1978 |
|  | XP\_065063860\_11043-model4.5 | -12.1978 |
|  | XP\_065063860\_11043-model4.6 | -12.1978 |
|  | XP\_065063860\_11043-model4.7 | -12.1978 |
|  | XP\_065063860\_11043-model4.8 | -12.1978 |
|  | XP\_065063860\_11043-model4.9 | -12.1978 |
|  | XP\_065063860\_11043-model4.10 | -12.1399 |
|  | XP\_065063860\_11043-model4.11 | -12.1399 |
|  | XP\_065063860\_11043-model4.12 | -12.1399 |
|  | XP\_065063860\_11043-model4.13 | -12.1399 |
|  | XP\_065063860\_11043-model4.14 | -12.1399 |
|  | XP\_065063860\_11043-model4.15 | -12.129 |
| # cluster 5 - Size: 2 |  |  |
|  | XP\_065063860\_11043-model5 | -12.1939 |
|  | XP\_065063860\_11043-model5.1 | -12.1475 |
| # cluster 6 - Size: 5 |  |  |
|  | XP\_065063860\_11043-model6 | -12.1113 |
|  | XP\_065063860\_11043-model6.1 | -12.1113 |
|  | XP\_065063860\_11043-model6.2 | -12.1113 |
|  | XP\_065063860\_11043-model6.3 | -12.1113 |
|  | XP\_065063860\_11043-model6.4 | -12.1113 |
|  |  |  |
